# A Case of Suspected Colocutaneous Fistula Within an Incisional Hernia Secondary to Sigmoid Diverticulitis in an Elderly Patient With Prior History of Cystectomy and Ileal Conduit Formation

**DOI:** 10.7759/cureus.103493

**Published:** 2026-02-12

**Authors:** Narayan Khanal

**Affiliations:** 1 General Surgery, Albury Wodonga Health, Albury, AUS

**Keywords:** colocutaneous fistula, complicated diverticulitis, incisional hernia, perforated diverticulitis, sigmoid diverticulitis

## Abstract

Diverticulitis is a common colonic pathology, but fistulous communication with the anterior abdominal wall is rare. We report a case of a suprapubic abscess with a radiologically suspected colocutaneous fistula arising from sigmoid diverticulitis in an 87-year-old man with prior cystectomy and ileal conduit formation. Laboratory investigations demonstrated significant inflammation, and contrast-enhanced computed tomography revealed a thick-walled suprapubic collection with a defined tract extending to an inflamed segment of sigmoid colon, consistent with a colocutaneous fistula. Management involved intravenous broad-spectrum antibiotics and ultrasound-guided percutaneous drainage, resulting in clinical improvement and resolution of sepsis. The patient was discharged with a plan for outpatient follow-up, and consideration of elective sigmoid resection should the fistula persist. This case highlights the importance of recognising atypical presentations of diverticulitis in patients with altered pelvic anatomy and underscores the role of cross-sectional imaging in diagnosis and guiding management.

## Introduction

Diverticulitis is a frequent cause of acute abdominal pain, particularly in older adults [1]. It arises from inflammation of colonic diverticula, most commonly in the sigmoid colon, and may be complicated by perforation, abscess, obstruction, or fistula formation [1].

Fistulas occur in approximately 2% of patients with diverticulitis, with the most common being colovesical and colovaginal [2]. Colocutaneous fistulas, particularly to the anterior abdominal wall, are uncommon [3]. Spontaneous colocutaneous fistulas arising from primary diverticular disease are rare and primarily reported as isolated case reports, whereas most enterocutaneous fistulas occur as postoperative or iatrogenic complications of abdominal surgery or intervention [4]. The pathophysiology involves chronic inflammation and microperforation of diverticula, which can extend to adjacent structures [5]. Previous pelvic surgery further distorts local anatomy, creating vulnerable planes for inflammatory tracts or abscesses to develop [6].

In patients who have undergone cystectomy and ileal conduit formation, postoperative adhesions and loss of the bladder barrier may predispose to unusual routes for fistula formation. We present a case of diverticulitis-associated suprapubic abscess within a hernia with a suspected sigmoid fistula in a post-cystectomy patient. The case highlights diagnostic challenges, radiological correlation, and the importance of recognising atypical presentations of diverticulitis in surgically modified pelvises. To the best of our knowledge, after a literature search, this is probably the first case of colocutaneous fistulisation into an incisional hernia following cystectomy.

## Case presentation

An 87-year-old male patient presented with a three-day history of suprapubic pain, swelling, and fever. He had loose bowel motions, but denied nausea, vomiting, or blood in stool. He had a past history of cystectomy and ileal conduit formation for high-grade urothelial carcinoma, performed 10 years ago.

On examination, he was febrile (39 °C), had a heart rate of 80 beats per minute, saturations of 100% on room air, and a respiratory rate of 16 breaths per minute. He had mild localised tenderness in the suprapubic region without obvious fluctuation. The ileal conduit appeared healthy and functional. Laboratory investigations revealed haemoglobin of 103 g/L, white cell count 16.6 ×10⁹/L, and C-reactive protein (CRP) 192 mg/L, consistent with acute infection and inflammatory response. The estimated glomerular filtration rate (eGFR) of 33 and creatinine of 158 were consistent with his baseline, and liver function tests were all within normal limits. He had chronic and stable anaemia compared with prior results, which was not felt to be contributory to the acute presentation or to require specific intervention during admission.

**Figure 1:**
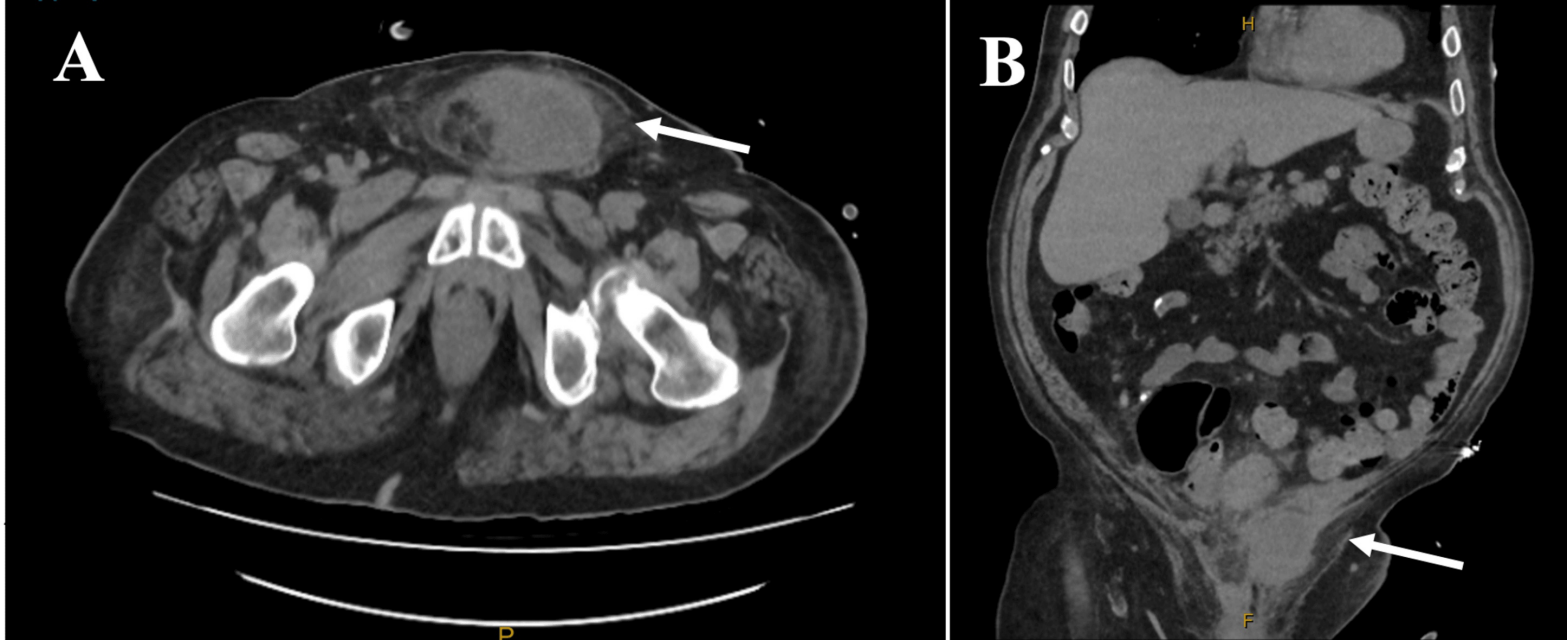
Contrast-enhanced CT of the abdomen and pelvis demonstrating a suprapubic abscess with suspected colocutaneous fistula secondary to sigmoid diverticulitis. (A) Axial CT image shows a thick-walled, peripherally enhancing suprapubic collection located slightly left of midline, abutting the dorsal margin of the penile base (white arrow). (B) Coronal CT image demonstrates a well-defined tract extending superiorly from the suprapubic collection to the inferior aspect of the sigmoid colon (white arrow), which shows associated mural thickening and pericolic fat stranding consistent with diverticulitis. No gas is seen within the collection, and there is no pneumoperitoneum, supporting a contained inflammatory fistula rather than free perforation.

Contrast-enhanced computed tomography (CT) with intravenous contrast revealed a thick-walled, enhancing suprapubic collection measuring 7.0 × 5.0 × 5.9 cm, slightly left of midline and abutting the dorsal margin of the penis. A well-defined 7 mm tract extended superiorly towards the sigmoid colon, suggestive of a fistulous connection. There was no gas within the collection, no pneumoperitoneum, and no contrast extravasation, consistent with a contained inflammatory fistula rather than free perforation. The sigmoid segment demonstrated mural thickening and pericolic fat stranding (Figure 1). Ultrasound confirmed a complex suprapubic collection (73 × 53 × 51 mm; volume 104 cc), partially within subcutaneous tissues and extending through the anterior abdominal wall fascia. The suspected fistulous tract adjacent to the collection measured approximately 8 mm in diameter.

The patient was commenced on intravenous piperacillin-tazobactam. Blood cultures were obtained on admission and showed no growth. Interventional radiology performed ultrasound-guided percutaneous drainage, yielding purulent fluid. Microbiology and cultures were sent for analysis. Clinical improvement was observed following drainage and antibiotic therapy, and inflammatory markers improved with treatment, with CRP decreasing to 13 mg/L and the white cell count improving to 11.0 ×10⁹/L at discharge. A plan was made for outpatient follow-up, with consideration of elective sigmoid resection should persistent fistulous communication be confirmed.

## Discussion

Diverticulitis is a common inflammatory disease of the colon, with prevalence increasing with age over 60 years [7]. While most cases remain uncomplicated and respond to conservative therapy, approximately 15% develop complications such as abscess, perforation, peritonitis, obstruction, or fistula formation. The sigmoid colon is the most frequent site of involvement due to increased intraluminal pressure and smaller diameter, resulting in a propensity for diverticular formation. The majority of diverticular fistulisation involves communication with adjacent hollow viscera, most notably colovesical and colovaginal tracts [8]. Colocutaneous fistulas, where the colon communicates with the skin or anterior abdominal wall, are exceedingly rare and often arise in the setting of previous surgery, radiotherapy, or chronic inflammatory states [9,10].

In this case, the patient’s prior cystectomy and ileal conduit formation likely played a role in the pathogenesis. Postoperative fibrosis, adhesions, and altered pelvic anatomy, as well as an incisional hernia, likely created a pathway of least resistance for an inflammatory or perforated diverticulum to track anteriorly. The bladder, which would normally act as a physical barrier between the sigmoid colon and the anterior abdominal wall, was absent, thereby allowing the diverticular inflammation to extend toward the suprapubic soft tissues. The result was a contained suprapubic abscess with a potential fistulous connection to the sigmoid colon, as demonstrated on CT imaging.

From a pathophysiological perspective, diverticulitis leading to colocutaneous fistula occurs when a walled-off pericolic abscess gradually erodes through adjacent fascial planes and soft tissues [11]. The anterior abdominal wall can become involved if pre-existing surgical planes, scarring, and hernial defects provide a route of extension [10]. Management of such cases should be individualised. Initial priorities include sepsis control with broad-spectrum intravenous antibiotics and radiologically guided drainage of any abscess cavity. In this case, the use of piperacillin-tazobactam and ultrasound-guided drainage was appropriate and effective, achieving clinical improvement. Non-operative management was chosen initially in this case due to the patient’s advanced age, clinical stability, absence of generalised peritonitis, and radiological features consistent with a contained inflammatory fistula rather than free perforation.

Initial control of sepsis with broad-spectrum antibiotics and image-guided drainage aligns with established management principles for complicated diverticulitis presenting with localised abscess formation. This approach allows stabilisation during the acute inflammatory phase and avoids the morbidity of emergency surgery in a high-risk patient. Potential long-term risks of conservative management include recurrent abscess formation, chronic fistulation, and the need to exclude underlying malignancy. For these reasons, interval follow-up and consideration of elective sigmoid resection remain important should fistulous communication persist or symptoms recur. In this case, elective surgical intervention was discussed as a contingency once acute inflammation had resolved. Definitive management depends on the persistence of the fistula. If the tract remains patent or recurrent abscesses develop, surgical resection of the involved sigmoid segment with primary anastomosis is generally recommended once the acute inflammatory phase has subsided [12].

This case also underscores the importance of considering diverticulitis as a differential diagnosis in any patient presenting with a suprapubic or lower abdominal wall abscess, particularly those with previous pelvic surgery. Key learning points include the potential for altered postoperative anatomy to result in atypical abscess locations and fistula pathways, the central role of cross-sectional imaging in establishing the diagnosis, and the value of individualised, stepwise management combining sepsis control with consideration of delayed definitive surgery. The clinical presentation may be atypical, as classical left lower quadrant pain or bowel symptoms can be absent. A high index of suspicion, combined with appropriate imaging, is therefore essential for diagnosis.

## Conclusions

This case highlights an unusual manifestation of sigmoid diverticulitis presenting as a suprapubic abscess with a suspected colocutaneous fistula in an elderly patient with a past history of cystectomy. The presence of prior pelvic surgery and altered anatomy can create atypical pathways for inflammatory extension, emphasising the need for heightened diagnostic vigilance when evaluating abdominal wall abscesses in this population. Prompt cross-sectional imaging, antibiotic therapy, and image-guided drainage form the cornerstone of initial management, with definitive surgical intervention reserved for persistent or recurrent disease.
